# Probing the Dual-Route Model of the SNARC Effect by Orthogonalizing Processing Speed and Depth

**DOI:** 10.1027/1618-3169/a000577

**Published:** 2023-04-11

**Authors:** Daniele Didino, Matthias Brandtner, Maria Glaser, André Knops

**Affiliations:** ^1^ Department of Psychology, Humboldt-Universität zu Berlin, Berlin, Germany; ^2^ Department of Psychology, Universität Potsdam, Potsdam, Germany; ^3^ Université Paris Cité, UMR 8240 LaPsyDÉ, CNRS, Paris, France

**Keywords:** SNARC effect, color task, response latency, semantic processing, dual-route model

## Abstract

**Abstract:** The dual-route model explains the SNARC (Spatial-Numerical Association of Response Codes) effect assuming two routes of parallel information processing: the unconditional route (automatic activation of pre-existing links) and the conditional route (activation of task-specific links). To test predictions derived from this model, we evaluated whether response latency in superficial number processing modulates the SNARC effect in a color task (participants judged the color of a number). In Experiment 1, participants performed a parity task, an easy color task (short RTs), and a difficult color task (RTs similar to those of the parity task). A SNARC effect emerged only in the parity task. In Experiment 2, participants performed a color task and a secondary task under four conditions chosen to orthogonally manipulate response latency (short vs. long) and processing depth (semantic vs. perceptual). Only the long-latency perceptual-processing condition elicited a SNARC effect. To explain these results, we suggest that the cognitive resources required by a secondary task might dilute the SNARC effect. Our results indicate that the dual-route model should be modified to take into account additional factors (e.g., working memory load) that influence the level of activation of the unconditional route.


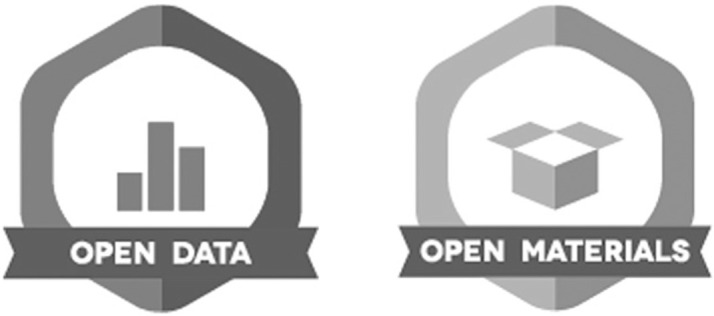


The Spatial-Numerical Association of Response Codes (SNARC) effect refers to the fact that relatively small and large numbers elicit faster left-sided and right-sided responses, respectively (Dehaene et al., 1993; for a review, see Fischer & Shaki, 2014; Van Dijck et al., 2015; Wood et al., 2008). The original explanation assumed that the effect arises from the congruency of response side and the position of the number on a spatially organized mental representation (Dehaene, 1997; Dehaene et al., 1993; Hubbard et al., 2005). This congruency emerges from cultural practices such as writing/reading direction (Göbel, 2015; Göbel et al., 2011, 2015; Shaki et al., 2009, 2012) and finger counting habits (Fischer, 2008; Hohol et al., 2022) and is linked to mathematical expertise (Cipora et al., 2016; Kramer et al., 2018).

The dual-route model is an alternative model that does not assume that the number representation is spatially organized (Gevers et al., 2006, 2010; Santens & Gevers, 2008; see also the polarity correspondence account, Proctor & Cho, 2006). This model proposes the existence of two routes of parallel information processing, in which numbers are coded into binary categories. The unconditional route is automatically activated, regardless of the task requirements, and classifies numbers as small or large based on their relative magnitude. Then, a pre-existing, culturally defined coding links magnitude categories (small vs. large) with spatial coordinates (left vs. right response sides). The conditional route defines a short-term stimulus–response mapping based on the task requirements. This route codes numbers into task-specific categories (e.g., even and odd in a parity task) and links them to spatial response coordinates (e.g., even-left and odd-right, or vice versa). The unconditional route (automatic activation of pre-existing links) and the conditional route (activation of task-specific links) can cooperate or compete during the response selection. The SNARC effect emerges from the congruency between these two routes. Response times are longer when the two routes activate opposite spatial responses and shorter when they converge on the same response side.

According to the dual-route model, the duration of the number processing is an important factor to influence the SNARC effect (Gevers et al., 2006). The longer the processing, the stronger the SNARC effect because the unconditional route has more time to interfere with response selection. Therefore, the dual-route model predicts that the strength of the SNARC effect increases along with response latency. The influence of response latency on the strength of the SNARC effect has also been confirmed in other studies (Cipora et al., 2019; Didino et al., 2019; Pressigout et al., 2019; Roettger & Domahs, 2015; Wood et al., 2008; Yu et al., 2020; but see Wood et al., 2006). Since the unconditional route is automatically activated regardless of the task requirements, the SNARC effect should not be modulated by the level of semantic processing required by the task. Therefore, tasks that imply semantic processing (e.g., magnitude or parity) should not generate a stronger effect compared to tasks that only require the processing of peripheral features (e.g., color).

In a previous study, we investigated whether the strength of the SNARC effect was modulated by the amount of semantic processing (Didino et al., 2019). Participants performed different tasks requiring different levels of semantic processing. The results showed that the strength of the SNARC effect was proportional to response latency and not influenced by the semantic processing required by the task. The study also included a color discrimination task in which participants judged the color of the font of the presented number. The color task had the shortest reaction times (RTs) and showed no evidence of a SNARC effect. However, when stratifying response latencies within the color task and analyzing only the longest RTs, a small (but nonsignificant) SNARC effect emerged in the color task. This underlines the idea that even in tasks where surface features of a number have to be processed (i.e., no deep semantic processing occurs), a SNARC effect potentially emerges under conditions that allow the unconditional route to interfere with the conditional route.

In previous studies, color tasks have repeatedly shown not to elicit a SNARC effect (Didino et al., 2019; Fias et al., 2001; Lammertyn et al., 2002). In two experiments with separate samples, Fias et al. (2001) manipulated the difficulty of two color tasks. Both an easy (fast RTs) color task and a difficult (slow RTs) color task showed no evidence of a SNARC effect. Fias and colleagues concluded that the color task does not elicit a SNARC effect because it does not rely on parietal resources and thus does not overlap with the processing of numerical information. Cleland and Bull (2019) studied under what conditions a SNARC effect can be found in a color task. They showed that a SNARC effect can be elicited (a) if participants recognize the stimulus as a number before response or (b) if the stimulus is viewed long enough to allow the number processing to interfere with the decision. These results suggest that both depth and duration of processing can independently elicit a SNARC effect.

The current study tests this idea by designing a color task with considerably longer RTs to test if a SNARC effect emerges under these conditions. Hence, the current study aimed to evaluate the dual-route model and the influence of response latency and semantic processing on the SNARC effect. If one or both of these factors allow generating a SNARC effect in a color task (hereafter, color SNARC), it will provide strong evidence for a relationship between the factors and the SNARC effect. According to the dual-route model, the SNARC effect should be primarily modulated by response latency, whereas the impact of semantic processing should be minimal or absent. On the other hand, if the SNARC effect is mainly influenced by semantic processing (with a weaker or no contribution from response latency), it would provide strong evidence against the model. This study included two experiments. In Experiment 1, participants performed a parity task, an easy color task (i.e., short RTs), and a difficult color task (long RTs). The difficult color task was designed to elicit approximately equal RTs compared to the parity task. According to the dual-route model, a color SNARC should emerge in the difficult color task due to the long RTs (similar to those of the parity task). In Experiment 2, participants performed a color task and a secondary task under four conditions chosen to manipulate response latency and the level of semantic processing required by the task. We expected the strength of the color SNARC to be affected by these two factors. However, we go beyond previous studies (Cleland & Bull, 2019; Fias et al., 2001) since we evaluated the influence of these two factors on the SNARC effect in a within-subject design.

## Experiment 1

Experiment 1 aimed to test the prediction of the dual-route model that the strength of the SNARC effect increases along with response latency because longer RTs should provide the unconditional route with more time to interfere with response selection. Participants performed a parity judgment task, an easy color task, and a difficult color task. The easy color task was associated with fast RTs, whereas the difficult color task was designed to generate long response latencies, comparable to those associated with the parity task. We considered the parity task as a benchmark for other tasks and expected to find a standard SNARC effect (e.g., Cipora et al., 2019; Dehaene et al., 1993; Didino et al., 2019). Due to its fast response latency, the easy color task should not elicit a SNARC effect (see also Didino et al., 2019; Fias et al., 2001; Lammertyn et al., 2002). In the difficult color task, given the long response latency, the unconditional route should have enough time to interfere with the response selection, and thus, we expected to find a SNARC effect.

### Methods

#### Participants

Twenty-eight participants took part in the study. The data of three participants were excluded from the analysis for having poor accuracy in at least one block (<70%). Therefore, we analyzed the data of 25 participants (18 female, 7 male; *M*_age_ (*SD*) = 31.2 (11.3), range = 18–54). All participants had normal or corrected-to-normal vision and no color-vision deficits and gave informed consent to participate in this experiment for course credits or 8€.

#### Stimuli, Tasks, and Design

Arabic digits ranging from 1 to 9, excluding 5, were used as target stimuli in all tasks. Each participant performed three tasks. In a parity judgment task, participants were asked to judge if the target was even or odd. In two color judgment tasks (easy and difficult), participants classified the color of the target. In the easy color task, the two colors were lavender blue (RGB: 204, 204, 255) and blue (RGB: 0, 0, 255). In the difficult color task, the two colors were light blue (RGB: 42, 42, 255) and blue (RGB: 0, 0, 255). All numbers were presented in both colors within the same block. The light blue RGB values of the difficult color task were selected, after running a pilot experiment with 11 participants, to have similar mean RTs in the parity and difficult color tasks.

The parity task was always performed first, and then, the order of the color tasks was counterbalanced across participants. Each task included two blocks, in which the response mapping was reversed and counterbalanced across participants (Table E1 in Electronic Supplementary Material, ESM 1). In total, participants performed six blocks (3 tasks × 2 response mappings). Each number was repeated 26 times in each block. Therefore, the total number of trials was 1,248 (8 digits × 26 repetitions × 6 blocks). Numbers were pseudorandomly presented with the constraint that the same digit could not be presented on two consecutive trials. To familiarize with the block-specific response mappings, 16 practice trials preceded each block in the parity task and in the easy color task (each number presented twice in a randomized order) and 48 practice trials in the difficult color task (each number presented six times in a randomized order). In the practice trials, both accuracy and speed feedback were provided (no feedback was presented during the test blocks).

#### Procedure

The same procedure was used in all tasks. Stimulus presentation and response collection were implemented in MATLAB using the Psychophysics Toolbox (Brainard, 1997; Kleiner et al., 2007; Pelli, 1997). Stimuli were presented in the center of the monitor and were 22 mm high and 15 mm wide. Participants sat at approximately 50 cm from the monitor (visual angle: 2.5° × 1.7°).

Each trial started with a fixation mark (#) presented for 500 ms, followed by the target, which remained on the screen until the response or for 1,300 ms. Participants were instructed to press a key on the left (“left-control” with the left hand) or right (“enter” on the numpad with the right hand) sides of the keyboard according to the block-specific instructions. The two keys were approximately 40 cm apart. Following the offset of the target, the next trial began after an intertrial interval of 500 ms consisting of a black screen. Except for the targets in the color tasks, all stimuli were printed in white against a black background.

A small sheet showing the response mapping was placed under the monitor to remind the participants of the block-specific instructions. Participants were asked to respond as fast and accurately as possible and could take short breaks between the blocks. The experiment lasted approximately between 40 and 60 min (average duration: 45 min).

### Results

**Figure 1:**
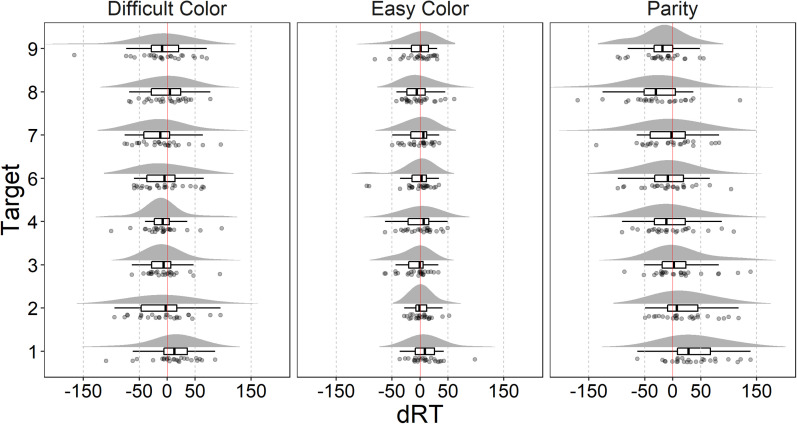
Separately for each target and task, dRT distributions are presented as density plot, boxplot, and raincloud plot (points represent participants' dRTs jittered along the *y* axis). Vertical solid lines mark 0 dRT, that is, no difference between the RT of the right and left hands.

Analysis^1^ was performed in R (R Core Team, 2021) and RStudio (RStudio Team, 2021). The data and the annotated R code used for the analysis are available at the Open Science Framework (Didino, 2023). Participants had a very high accuracy in all tasks (parity judgment: *M* = 0.94, *SD* = 0.04; easy color task: *M* = 0.97, *SD* = 0.03; difficult color task: *M* = 0.92, *SD* = 0.05). Accuracy data are likely affected by a ceiling effect and thus will not be further analyzed. Trials with incorrect (1787 trials, 5.73%) or omitted responses (158 trials, 0.51%) or RTs faster than 250 ms (29 trials, 0.09%) were excluded from the analysis. For each participant and each task, correct trials with RTs more than 3 *SD* from the mean were considered outliers and excluded from the analysis (145 trials, 1.48%, for parity task; 156 trials, 1.55%, for easy color task; 157 trials, 1.65%, for difficult color task). Mean RTs (in ms) across tasks are reported in Table 1. To measure the SNARC effect, we calculated the dRT as mean RTs for the right hand minus mean RTs for the left hand, separately for each target number, task, and participant (Fias et al., 1996; see also Pinhas et al., 2012; Tzelgov et al., 2013). The distributions of dRTs are reported in Figure 1.

**Table 1:** Mean and standard deviation (*SD*) for RTs and estimated central tendencies, 2.5 (Q2.5) and 97.5 (Q97.5) quantiles, and Bayes factors for the posterior distributions of slope coefficients

Task	RTs		Slope				
	Mean	*SD*	Estimate	Q2.5	Q97.5	BF_01_	BF_10_
Parity	553	74	−6.67	−9.76	−3.52	0.01	180
Easy color	466	51	−0.96	−2.75	0.85	6.30	0.16
Difficult color	576	71	−1.16	−2.90	0.60	4.60	0.22
Color tasks comparison	—	—	−0.20	−2.69	2.29	11	0.09
*Note*. Color tasks comparison refers to the difference between the difficult and easy color tasks. BF_01_ = evidence in favor of the null hypothesis; BF_10_ = evidence in favor of the alternative hypothesis.							

The dRTs were analyzed with a hierarchical Bayesian model^2^ with normal likelihood function. The priors and the other specifications are reported in annotated R code in the OSF deposit (Didino, 2023). The model likelihood was defined as follows:

dRT∼Normal(μ,σ) 1

where

μ=0+(task/target_c)+(0+(task/target_c)|sj) 2

**Figure 2:**
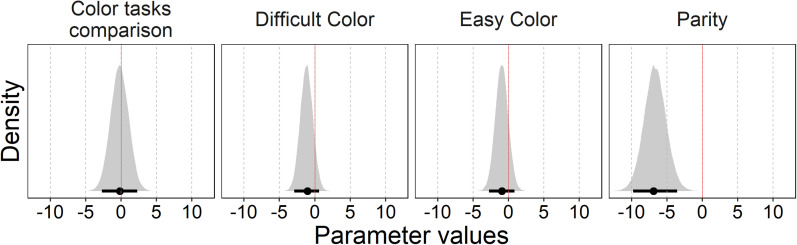
Posterior distributions and 95% highest-density intervals (HDI, black bar under the distribution) for slopes in the three tasks. Color tasks comparison refers to the difference between the difficult and easy color tasks.

The variable *task* includes three levels (parity, color difficult, color easy). The variable *target_c* is the centered version of the variable target and assumes the values −4, −3, −2, −1, 1, 2, 3, 4 (corresponding to 1, 2, 3, 4, 6, 7, 8, 9 in the noncentered variable). The formula for μ has no intercept, and target is set as nested in task. In other words, the model estimated a separate intercept and slope for each task. The variable *sj* represents the participant code. Here, we only discuss the coefficients for slope, which are relevant for the hypothesis testing, whereas the other coefficients are reported in the OSF. The slopes were interpreted as a measure of the SNARC effect, with larger negative values corresponding to a stronger effect. Table 1 and Figure 2 summarize the posteriors of the coefficients for slope.

We tested the null hypothesis that the coefficient for the slope was not different from zero (H_0_: β_parity:target_c_ = 0; β_color_easy:target_c_ = 0; β_color_difficult:target_c_ = 0). The alternative hypothesis was two-sided (H_1_: β_parity:target_c_ ≠ 0; β_color_easy:target_c_ ≠ 0; β_color_difficult:target_c_ ≠ 0). The prior distribution for slope was specified as a normal distribution with μ = 0 and σ = 10 (see the OSF). Hypotheses were tested with the *hypothesis* function from the R Package *brms*. Bayes factors were computed via the Savage–Dickey density ratio method (i.e., the posterior density at the point of interest, here zero, divided by the prior density at the same point). Bayes factors and credible intervals are reported in Table 1. There was strong evidence for a SNARC effect in the parity task. We found a BF_10_ > 100, which indicates that the observed data were more than 100 times more likely under H_1_ than H_0_. For both color tasks, there was moderate evidence of a lack of color SNARC. We found a BF_01_ > 4, which indicates that the observed data were more than four times more likely under H_0_ than H_1_. For the comparison between the two color tasks (difficult color task minus easy color task), there was strong evidence that the two tasks did not differ. We found BF_01_ = 11, which indicates that the observed data were more than 10 times more likely under H_0_ than H_1_.

**Figure 3:**
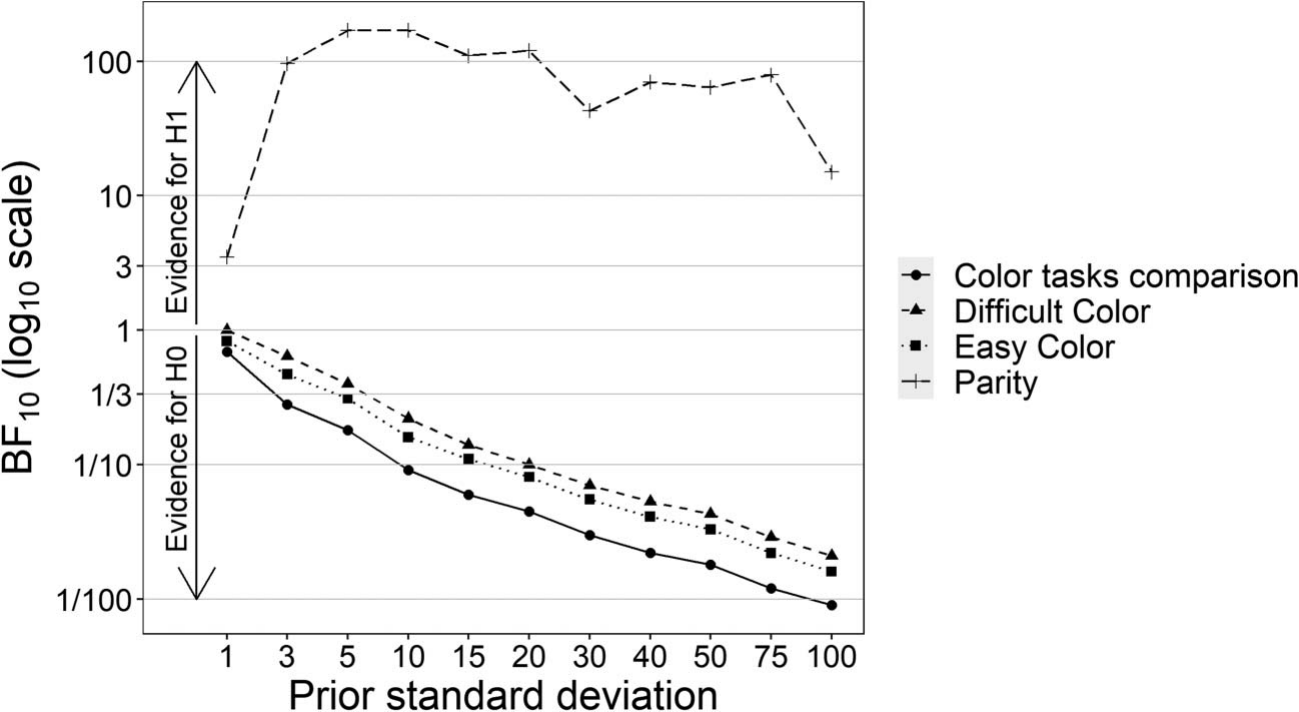
Bayes factors for H_1_ (BF_10_) across standard deviations of the prior distributions as a function of task. The *y*-axis is in log_10_ scale. Horizontal lines represent the decision criteria for the Bayes factor interpretation (i.e., 1–3 anecdotal evidence, 3–10 moderate evidence, 10–100 strong evidence, >100 decisive evidence).

Since Bayes factors are strongly influenced by the prior distribution, we also performed a sensitivity analysis on the effects of interest. Bayes factors were calculated for different *SD*s for the priors related to the slope coefficients. We used the *SD*s 1 (strongly informed prior), 3, 5, 10, 15, 20, 30, 40, 50, 75, and 100 (very generic broad prior). The results are reported in Figure 3. Except for the extreme and highly implausible values 1 and 100, the Bayes factors BF_10_ for the parity task are relatively stable and those for the color tasks and their comparison decrease regularly.

### Discussion

Experiment 1 aimed to evaluate whether long response latencies, comparable to that of a parity task, can elicit a SNARC effect in a color task that capitalizes on non-numerical surface features of the stimulus (i.e., font color) and hence is not processed via a dorsal pathway (Fias et al., 2001). The results showed that mean RTs were compatible between the parity and difficult color tasks, whereas they were faster in the easy color task. Bayesian factors provided strong evidence for a SNARC effect in the parity task and moderate evidence of a lack of SNARC effect in both color tasks. A sensitivity analysis confirmed that these Bayes factors remained relatively stable across various prior distributions.

According to the dual-route model, long response latencies should allow the unconditional route to interfere with the decision. However, despite the long RTs, the difficult color task did not elicit a SNARC effect. The lack of color SNARC might indicate that the activation of the unconditional route in the dual-route framework is not as unconditional as previously thought. It might be activated only under certain conditions. In accordance with recent findings, a minimal level of dorsal load is necessary for the SNARC effect to emerge (Cleland & Bull, 2019). Only under this condition, the unconditional route interferes with the decision. In our experiment, the difficult color task required only the discrimination of peripheral, nonsemantic stimulus features, and thus, the level of number processing might be too low to activate the unconditional route. In Experiment 2, we modulated the depth and the duration of the number processing to evaluate whether these factors can elicit a color SNARC.

## Experiment 2

According to the dual-route model, a long response latency should generate a SNARC effect because it provides the unconditional route with enough time to interfere with response selection. However, Experiment 1 showed that long latency alone was not enough to elicit a color SNARC. Experiment 2 investigated whether the combined effect of semantic processing and long response latency can evoke a color SNARC. Participants performed a color judgment task and a secondary task, which required processing semantic (parity) or perceptual features (font style) of the stimulus. The instruction on which feature participants should base their decision on was indicated by a cue that appeared before (short latency) or after (long latency) the target number. These four conditions (semantic long-latency, semantic short-latency, perceptual long-latency, and perceptual short-latency) allowed the orthogonal manipulation of the amount of semantic processing required by the task and the duration of the interval between loading the number in working memory and selecting a response. Based on the dual-route model and on the additional hypothesis that a minimum level of semantic number processing is required to elicit a color SNARC, we formulated the following predictions. In the two short latency conditions, regardless of the required processing (semantic vs. perceptual), we expected no color SNARC because the fast response latency does not allow the unconditional route to interfere with the response selection. The perceptual processing long-latency condition could generate a color SNARC because the unconditional route could have enough time to interfere with the decision. However, the strongest color SNARC was expected in the long-latency condition with semantic processing because the task requires deeper number processing and the unconditional route has enough time to interfere with the decision.

### Methods

#### Participants

Thirty-two participants took part in the study (22 female, 9 male, 1 reported being not represented by these two categories; mean age (*SD*) = 23.2 (4.3), range = 18–36). All participants had normal or corrected-to-normal vision and no color-vision deficits and gave informed consent to participate for course credits.

#### Stimuli, Task, and Design

Arabic digits 1, 2, 8, and 9 were used as the target in all conditions. Participants performed four blocks. Each block included a color task and one of two secondary tasks. In the color task trials, participants classified the color of the target. The two colors were blue (RGB: 0, 0, 255) and green (RGB: 0, 128, 0). All numbers were presented in both colors within the same block. In the two secondary Go/No-Go tasks, the participants were asked to judge either the parity (parity task) or the font style (regular vs. bold font; font task) of the target.

**Figure 4:**
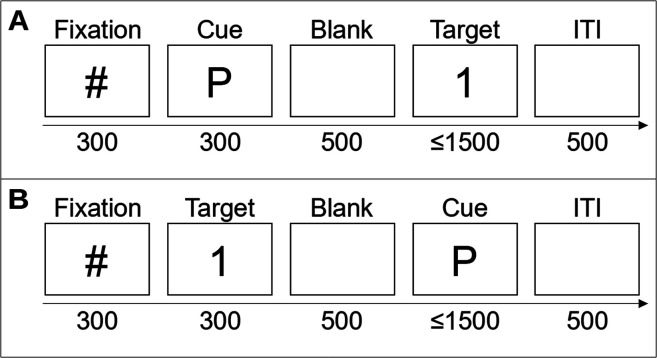
Trial structure of Experiment 2 for cue-first trials (A) and target-first trials (B). The numbers below the arrow represent the duration of the stimuli in milliseconds. The label above the boxes represents the stimuli (ITI: intertrial interval).

Two variables were orthogonally manipulated: secondary task and cue–target order. The secondary task was the parity task in two blocks and the font task in the other two blocks. Since all blocks included the color task, in what follows, we will only refer to the secondary task (parity vs. font). The cue could be presented before (cue-first, i.e., short-latency condition) or after (target-first, i.e., long-latency condition) the target (see Figure 4). Therefore, the four blocks were parity cue-first, parity target-first, font cue-first, and font target-first. The order of the blocks was counterbalanced across participants with the following restrictions. The same secondary task was presented in Blocks 1–2 and 3–4, and the cue–target order was the same in Blocks 1–3 and 2–4 (see Table E2 in ESM 1).

For the color task, each target-color combination was repeated 15 times in each block (15 repetitions × 4 targets × 2 colors = 120 trials). Each block also included 40 trials for the secondary task (i.e., 25% of the total trials of the block). Therefore, each block included 160 trials (120 color task trials + 40 secondary task trials), and the total number of trials was 640 (160 trials × 4 blocks). Targets were randomly presented. To familiarize with the block-specific response mapping, each block started with 20 practice trials (14 color task trials + 6 secondary task trials), randomly selected from the stimulus set. In the practice trials, both accuracy and speed feedback were provided (no feedback was presented during the test blocks).

#### Procedure

Stimulus presentation and response collection were implemented in PsychoPy (Peirce et al., 2019) using the Pavlovia platform for online experiments. Since the experiment was conducted online, we do not know the height and width of the stimuli and the distance of the participant from the monitor. Stimuli were presented in the center of the monitor. In each trial, a visual cue informed the participant which task was to be performed (“C” for color, “P” for parity, and “F” for font). In the cue-first condition, each trial started with a fixation mark (#) presented for 300 ms, followed by a cue (500 ms), a blank (500 ms), and a target, which remained on the screen until the response or for 1,500 ms (Figure 4). Participants were instructed to keep the left and right index fingers on the “X” and “M” keys of the keyboard, respectively. In the color task, one-half of the participants pressed “X” for green and “M” for blue, and vice versa for the other half. In the secondary task, participants were instructed to press both “X” and “M” in Go trials or not press any key in No-Go trials. The mapping between response (Go vs. No-Go) and stimulus feature (bold vs. no-bold in the font task, odd vs. even in the parity task) was counterbalanced across participants (see Table E3 in ESM 1). Participants were instructed to remember the cue and to respond immediately after the onset of the target. Following the offset of the target, the next trial began after an intertrial interval of 500 ms consisting of a black screen. Except for targets, all stimuli were printed in white against a black background. In the target-first condition, the cue–target order was reversed, but every other aspect of the procedure was the same. Participants were instructed to remember the target and to respond only after the onset of the cue.

Before each block, participants performed a short practice block (20 trials) with the online supervision of the experimenter. Participants were asked to respond as fast and accurately as possible and could take short breaks between the blocks. An experimental session lasted between 30 and 45 min.

### Results

Analysis was performed in R (R Core Team, 2021) and RStudio (RStudio Team, 2021) using the same packages as in Experiment 1. In the color task, the mean accuracy was 0.95 (*SD* = 0.05) in parity cue-first, 0.92 (0.07) in parity target-first, 0.94 (0.06) in font cue-first, and 0.93 (0.06) in font target-first. Accuracy data are likely affected by a ceiling effect and thus will not be further analyzed. For the secondary task, the accuracy was lower and with higher variance (see the “exp2_1_preprocessing” file in the OSF deposit for more details). Most of the participants performed above 0.75. Some participants had accuracy below 0.25, indicating that they probably inverted the mapping between Go and No-Go trials. Seven participants had accuracy between 0.4 and 0.7 in the first block (two participants also in the second block). The secondary tasks have not been further analyzed because they were only used to encourage the participants to process additional features of the stimulus (i.e., parity or font). The data and the R code used for the analysis are available at the Open Science Framework (Didino, 2023).

**Figure 5:**
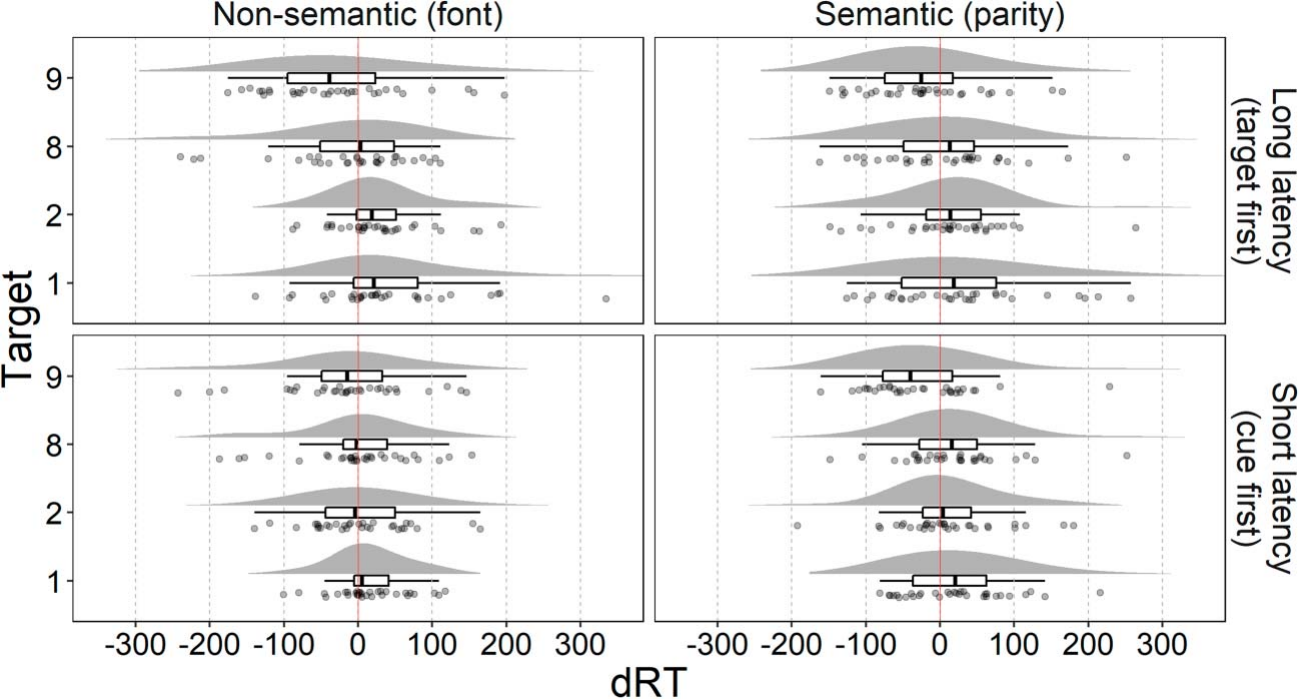
Separately for each target and condition, dRT distributions are presented as density plot, boxplot, and raincloud plot (points represent participants’ dRTs jittered along the *y*-axis). Vertical solid lines mark 0 dRT, that is, no difference between the RT of the right and left hands.

The following analyses were performed on the color task trials only. Trials with incorrect (1,042 trials, 6.78%) or omitted responses (400 trials, 2.6%), or RTs faster than 250 ms (77 trial, 0.5%) were excluded from the analysis. For each participant and each condition, correct trials with RTs more than 3 *SD* from the mean were considered outliers and excluded from the analysis (53 trials, 1.46%, for parity cue-first; 30 trials, 0.86%, for parity target-first; 55 trials, 1.53%, for font cue-first; 30 trials, 0.84%, for font target-first). Mean RTs (in ms) across conditions are reported in Table 2. Separately for each target, condition, and participant, the dRTs were calculated as mean RTs for the right hand minus mean RTs for the left hand (Fias et al., 1996; see also Pinhas et al., 2012; Tzelgov et al., 2013). The distributions of dRTs are reported in Figure 5.

**Table 2:** Mean and standard deviation (SD) for RTs and estimated central tendencies, 2.5 (Q2.5) and 97.5 (Q97.5) quantiles, and Bayes factors for the posterior distributions of slope coefficients

Task	RTs		Slope				
	Mean	*SD*	Estimate	Q2.5	Q97.5	BF_01_	BF_10_
Parity cue-first	580	95	−3.27	−6.97	0.43	1.20	0.86
Parity target-first	655	114	−4.05	−8.64	0.57	0.92	1.10
Font cue-first	588	107	−2.65	−6.44	1.16	2.10	0.48
Font target-first	627	108	−7.50	−11.88	−3.04	0.02	36.00

*Note*. BF_01_ = evidence in favor of the null hypothesis; BF_10_ = evidence in favor of the alternative hypothesis.

The dRTs were analyzed with a hierarchical Bayesian model^3^ with a normal likelihood function. The priors and the other specifications are reported in annotated R code in the OSF. The model likelihood was defined as follows:

dRT∼Normal(μ,σ) 3

where

μ=0+(condition/target_c)+(0+(condition/target_c)|sj) 4

**Figure 6:**
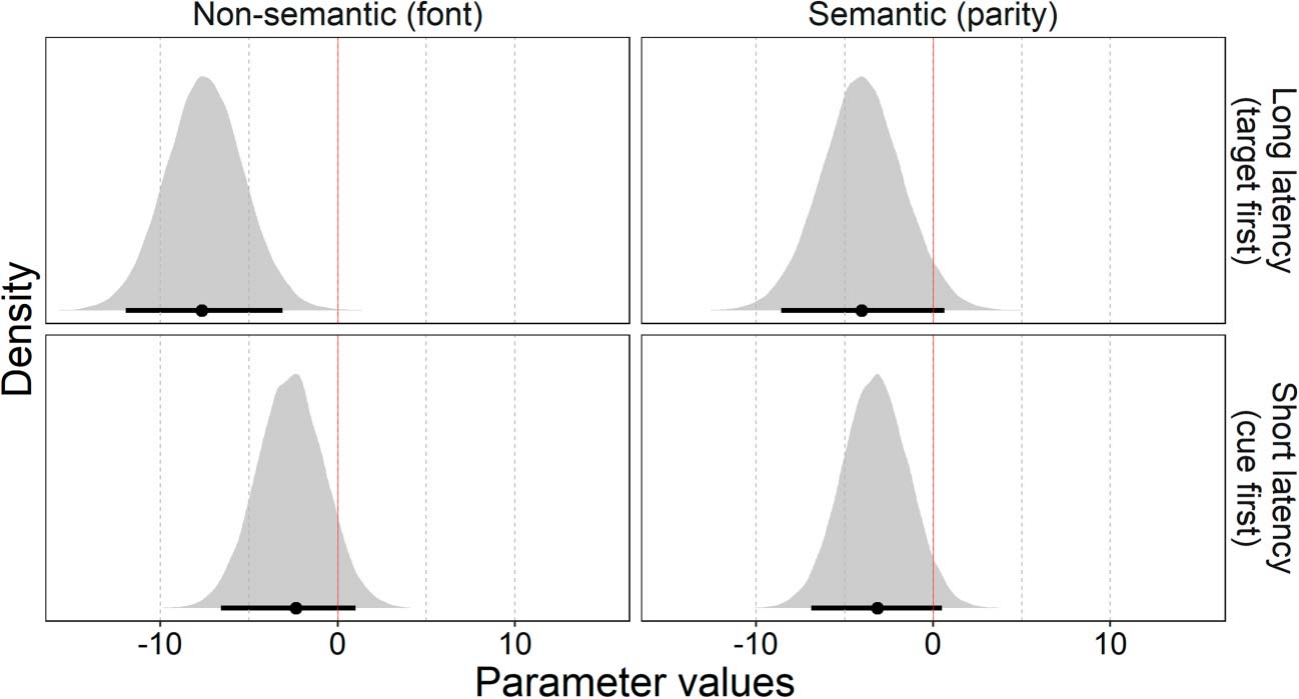
Posterior distributions and 95% highest-density intervals (HDI, black bar under the distribution) for slopes of the four conditions.

The variable *condition* includes four levels (parity cue-first, parity target-first, font cue-first, and font target-first). The variable *target_c* is the centered version of the variable target and assumes the values −4, −3, 3, 4 (corresponding to 1, 2, 8, 9 in the noncentered variable). The formula for μ has no intercept, and target is set as nested in condition. Therefore, the model estimated a separate intercept and slope for each condition. The variable *sj* represents the participant code. Here, we only discuss the coefficients for the slopes, which are relevant for the hypothesis testing (the other coefficients are reported in the OSF). The slopes were interpreted as a measure of the SNARC effect, with larger negative values corresponding to a stronger effect. Table 2 and Figure 6 report the summary for the posteriors of the coefficients for the slopes.

We tested the null hypotheses that the coefficients for the slopes were no different from zero (H_0_: β_parity__cue_first:target_c_ = 0; β_parity__target_first:target_c_ = 0; β_font__cue_first:target_c_ = 0; β_font__target_first:target_c_ = 0). The alternative hypotheses were two-sided (H_1_: β_parity__cue_first:target_c_ ≠ 0; β_parity__target_first:target_c_ ≠ 0; β_font__cue_first:target_c_ ≠ 0; β_font__target_first:target_c_ ≠ 0). The prior distribution for slopes was specified as a normal distribution with μ = 0 and σ = 10 (see OSF). Hypotheses were tested with the *hypothesis* function from the R package *brms*. Bayes factors were computed as in Experiment 1 and are reported in Table 2. There was strong evidence for a color SNARC in the font target-first condition. We found a BF_10_ = 36, which indicates that the observed data were 36 times more likely under H_1_ than H_0_. For the other conditions, we found BF_10_ < 2 (BF_01_ < 3), which indicates inconclusive evidence for either H_0_ or H_1_.

**Figure 7:**
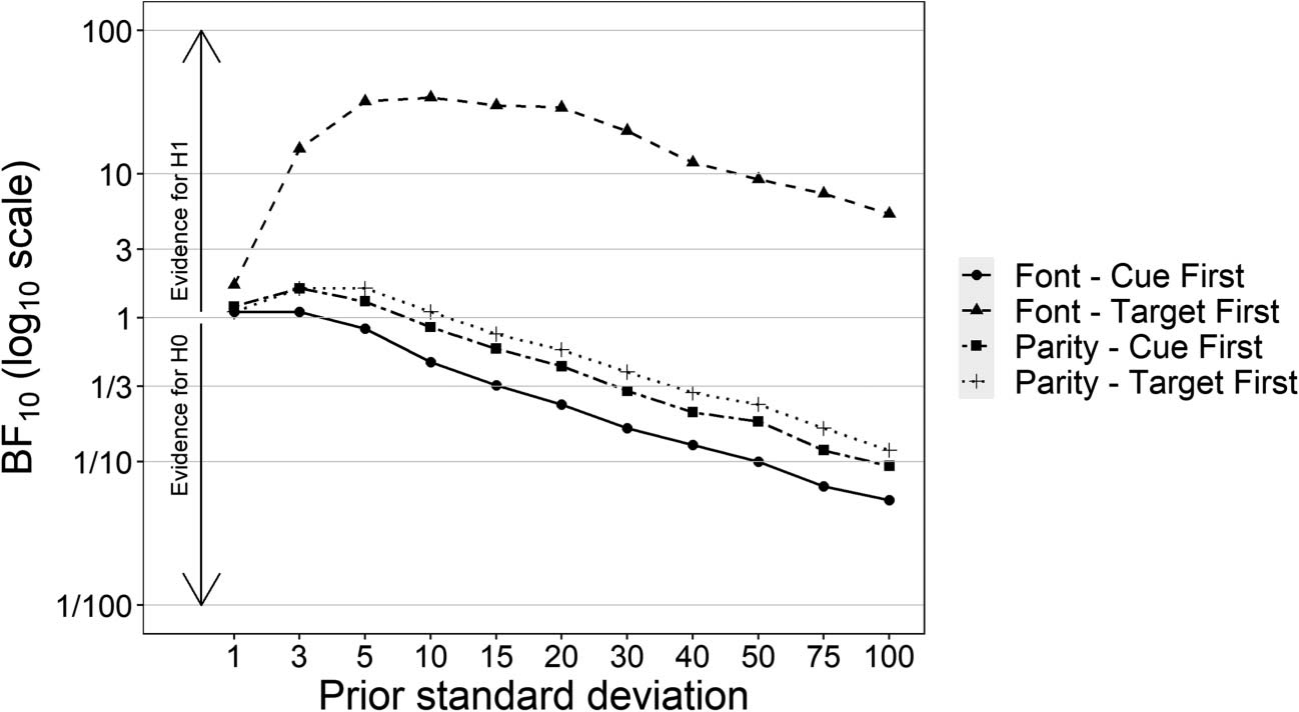
Bayes factors for H_1_ (BF_10_) across standard deviations of the prior distributions as a function of condition. The *y*-axis is in log_10_ scale. Horizontal lines represent the decision criteria for the Bayes factor interpretation (i.e., 1–3 anecdotal evidence, 3–10 moderate evidence, 10–100 strong evidence, >100 decisive evidence).

Since Bayes factors are strongly influenced by the prior distribution, we also performed a sensitivity analysis on the effects of interest. Bayes factors were calculated for different *SD*s for the priors related to the slope coefficients. We used the *SD*s 1 (strongly informed prior), 3, 5, 10, 15, 20, 30, 40, 50, 75, and 100 (very generic broad prior). The results are reported in Figure 7. Except for the extreme and highly implausible values 1 and 100, the Bayes factors BF_10_ for the font target-first condition are relatively stable (approximately above 10) and those for the other conditions decrease regularly.

### Discussion

Experiment 2 aimed to elicit a SNARC effect in a color task by manipulating response latency and semantic processing. Bayes factors provided strong evidence for a color SNARC in the font target-first condition and inconclusive evidence for either H_1_ or H_0_ in the other three conditions. A sensitivity analysis confirmed that these Bayes factors remained relatively stable across various prior distributions.

The color SNARC in the font target-first condition is consistent with the dual-route model, which predicts the strength of this effect is proportional to response latency. However, it is unclear why the effect did not emerge also in the parity target-first condition. This result may be related to the different degrees of cognitive resources required in these two conditions. This explanation is addressed in the “General Discussion” section.

An important role of the secondary task was to activate semantic (parity) or perceptual information (font) in working memory. However, some participants had low performance in the secondary task. Participants with very low accuracy (<0.25) have probably inverted the Go and No-Go responses. Seven participants had accuracy between 0.4 and 0.7. Since these participants did not perform the secondary task correctly, the required stimulus features (i.e., parity or font style) might not have been loaded in working memory. However, the low accuracy affected the first block and also the second block for only two participants (see the “exp2_1_preprocessing” file in the OSF). Therefore, these participants might have misunderstood the response procedure (i.e., key mapping or response windows) in the first block but correctly extracted the stimulus features.

## General Discussion

The dual-route model challenged the original explanation that the SNARC effect emerges from a spatially organized number representation (Dehaene, 1997; Dehaene et al., 1993; Hubbard et al., 2005). Compared to the classical view, the dual-route model had the advantage to account for various findings related to the SNARC effect, such as being located at the response selection stage, increasing along with response latency, and being relative to the numerical interval used in the experiment (see Gevers et al., 2006). Moreover, the model was able to predict a new feature of the SNARC effect (i.e., it has a categorical shape in magnitude tasks and a continuous shape in nonmagnitude tasks, e.g., parity judgment; Gevers et al., 2006), which was experimentally confirmed (e.g., Didino et al., 2019). The present study aimed to evaluate the dual-route model and to test whether a SNARC effect can be elicited in a color task by manipulating response latency and the amount of processing required by the task. The color task was chosen because it usually does not generate a SNARC effect (Cleland & Bull, 2019; Didino et al., 2019; Fias et al., 2001; Lammertyn et al., 2002). Experiment 1 tested whether long latencies suffice to elicit a color SNARC. Participants performed a parity task, an easy color task (fast RTs), and a difficult color task (RTs comparable to that of the parity task). According to the dual-route model (Gevers et al., 2006, 2010; Santens & Gevers, 2008), in the difficult color task, the long RTs should provide the unconditional route with enough time to interfere with the response selection. However, a SNARC effect emerged only in the parity task. Cleland and Bull (2019) suggested that the SNARC effect might be less automatic than previously assumed. Therefore, the lack of color SNARC might be because both a minimal amount of numerical processing (to activate the unconditional route) and long response latency (to interfere with the response selection) are needed to elicit an effect. Experiment 2 was designed to test this idea. Participants performed a color task together with a secondary task. Processing depth and stimulus processing time were orthogonally manipulated in four conditions: parity cue-first (semantic processing and short latency), parity target-first (semantic processing and long latency), font cue-first (perceptual processing and short latency), and font target-first (perceptual processing and long latency). Although the combined effect of semantic processing and long latency should have elicited a color SNARC in the parity target-first condition, a color SNARC emerged only in the font target-first condition. It is worth noting that a color task usually does not generate a SNARC effect. This result suggests that the level of semantic processing does not contribute significantly to the strength of the SNARC effect. Based on the dual-route model and on previous studies (Cipora et al., 2019; Didino et al., 2019; Pressigout et al., 2019; Roettger & Domahs, 2015; Wood et al., 2008; Yu et al., 2020; but see Wood et al., 2006), one would expect response latency to be the most important factor in eliciting a SNARC effect. Independent of the amount of semantic processing, long latency should allow the number processing to interfere with the response selection and thus evokes a color SNARC (see Cleland & Bull, 2019). However, Experiment 1 suggests that long latency alone is not enough to elicit a color SNARC, and in Experiment 2, only one of the two long-latency conditions generated a color SNARC. These results are difficult to explain within the framework of the dual-route model. Factors hitherto not accounted for by the dual-route model might have contributed to these results. In fact, if long latency is a determinant factor to evoke the SNARC effect, the relevant question becomes why the effect did not also emerge in the parity target-first condition, which also involved long latencies. One possibility is that the parity and font conditions differed in the level of cognitive resources required to perform the task.

In Experiment 2, participants had to switch between two tasks. The parity conditions required to switch between the processing of semantic (parity) and perceptual information (color), whereas the font conditions involved only the processing of perceptual features (font style and color). Switching between tasks requiring similar processing (i.e., perceptual information in the font target-first condition) and between tasks requiring processing different properties (i.e., semantic and perceptual information in the parity target-first condition) could rely upon different amounts of cognitive resources. Fias and van Dijck (2016) suggested that an essential requisite for eliciting a SNARC effect is that working memory resources must be available and that under working memory load the effect is reduced. Cognitive control can also modulate the SNARC effect (Gökaydin et al., 2018; Moro et al., 2018; Notebaert & Verguts, 2008; Pfister et al., 2013; Schliephake et al., 2022; Tan & Dixon, 2011; Wendt et al., 2013; Zhang et al., 2021). For example, Moro et al. (2018) found that the SNARC effect is modulated by repeating or switching key mapping compared to the previous trial. Moreover, task switching is particularly demanding in a parity task compared to magnitude judgment (Petruo et al., 2019). Switching between parity and color tasks could have required more working memory resources or higher cognitive control than switching between font and color tasks. Therefore, the lack of color SNARC in the parity target-first condition might be due to the higher cognitive resources used to switch between tasks or to perform the secondary task. The higher cognitive resources required in the parity target-first condition could have diluted the SNARC effect. Our study was not designed to evaluate how task switching influences the color SNARC, and thus, there were not enough trials to evaluate this hypothesis. However, we repeated the analysis of Experiment 2 including only task-repetition trials (i.e., color task trials preceded by a color task trial) and found similar results (the analysis is reported in OSF). Although this suggests that our results were not due to the influence of rule-switch trials, it should be taken into account that our experiment was not designed to evaluate the effects of task-switching and thus could be not sensitive enough to measure its effects. Future studies should include more secondary task trials to evaluate the effects of rule-switching and rule-repetition on the SNARC effect. Our results suggest that the kind of secondary task could also influence the SNARC effect. Therefore, future studies could include a larger set of secondary tasks to evaluate to what extent the amount of cognitive resources required by the secondary task can modulate the color SNARC.

Our results suggested that the SNARC effect is not influenced by the amount of semantic processing required by the task. However, our findings are difficult to explain within the framework of the dual-route model. The results of Experiment 1 are not in line with the predictions of the model. Similar RTs should have generated comparable SNARC effects in the parity and difficult color task. Experiment 2 indicated that the kind of processing and the amount of cognitive resources required by a secondary task can dilute the SNARC effect and influence the activation of the unconditional route. Overall, our results indicate that the activation of the unconditional route is not as unconditional as previously thought and that the dual-route model needs to be modified to take into account additional factors (e.g., working memory load) that could influence the activation unconditional route.

A limitation of this study refers to the performance in the secondary task in Experiment 2. For some participants, the performance in the secondary task was low in the first or second block. We assumed that this low performance was due to a misunderstanding of the response procedure and that participants correctly loaded in working memory required stimulus features. However, this could have affected the results. Future studies are required to confirm that our results were not affected by the low performance.

## Electronic Supplementary Material

The electronic supplementary material is available with the online version of the article at https://doi.org/10.1027/1618-3169/a000577

**ESM 1**. Model likelyhood; Figures (comparison between the observed data and simulated data) and supplementary Tables E1, E2, and E3.

## Footnotes

^1^
We used the following open-source packages: bayesplot (Gabry & Mahr, 2022), brms (Bürkner, 2017, 2018, 2021), broom (Robinson et al., 2022), ggpubr (Kassambara, 2020), here (Müller, 2020), janitor (Firke, 2021), knitr (Xie, 2014, 2015, 2021), kableExtra (Zhu, 2021), lme4 (Bates et al., 2015), magrittr (Bache & Wickham, 2020), plotly (Sievert, 2020), posterior (Bürkner et al., 2022), tidybayes (Kay, 2022), and tidyverse (Wickham et al., 2019). The raincloud plots were generated with the code from Allen et al. (2021).

^2^
We also examined whether the model misfit the by-subject error. We compared the posterior distribution of the standard deviation and the observed standard deviation for each participant. The results showed that the observed standard deviation of some participants was not a likely value in the posterior standard deviation. Therefore, we also ran a distributional model in which sigma (i.e., likelihood standard deviation) was allowed to vary across participants. The parameter estimation and the Bayes factor provide results similar to those obtained with the nondistributional model. See OSF for more details. The reasons for the choice of a normal distribution are reported in ESM 1. Frequentist analyses (i.e., *t*-tests) are included in OSF.

^3^
Also for Experiment 2, the observed standard deviation of some participants was not a likely value in the posterior standard deviation. Therefore, we ran a distributional model in which sigma (i.e., likelihood standard deviation) was allowed to vary across participants. The parameter estimation and the Bayes factor provide results similar to those obtained with the nondistributional model. See OSF for more details. Frequentist analyses (i.e., *t*-tests) are included in OSF.

## Notes

**Publication Ethics:** All participants gave informed consent to participate for course credits or 8€ (Experiment 1) or for course credits (Experiment 2). The study was approved by the Ethics Committee at the Department of Psychology of Humboldt-Universität zu Berlin (No. 2019-42).

**Open Data:** The electronic supplementary material (Tables E1–E3) is available with the online version of the article. Moreover, the raw and processed data and the annotated R code used for the analysis are freely accessible at the Open Science Framework. For access see, https://osf.io/5te7n/?view_only=dd50e38b6ec64d55b6125a22a2c22ee7 (Didino, 2023).
