# Supplementary material for: Probing the Dual-Route Model of the SNARC Effect by Orthogonalizing Processing Speed and Depth
Source: Exp Psychol. 2023 Apr 11;70(1):1–13. doi: 10.1027/1618-3169/a000577 (PMC10388237; doi:10.1027/1618-3169/a000577)
Supplement: Supplementary file 1 [file zea_70_1_1_esm1.pdf]

https://doi.org/10.1027/1618-3169/a000577

Model likelihood

The choice of a normal distribution as the model likelihood for the dRT is due to the difference between RT and dRT distributions. While the RTs are usually log-normally distributed (i.e., log(RT) is approximately normally distributed), in the analysis of the SNARC effect the dRTs are typically assumed to be approximately normally distributed. Following the standard procedure, we calculated the dRTs as mean RTs for the right hand minus mean RTs for the left hand. This was done separately for each participant and target number, and also for task (experiment 1) or condition (experiment 2). The dRTs are thus the results of a difference between means. Then the dRTs are used as the dependent variable and can be expressed by the formula:

$$dRT = \beta_0 + \beta_1 \times target + \varepsilon$$

Where: β_0 is the intercept, β_1 is the slope, and ε is the residual. In SNARC studies, the residual (ε) is usually assumed to be normally distributed. Therefore, given the normal distribution of the residual, we expressed the likelihood function as

$$dRT \sim Normal(u, \sigma)$$

Where, in experiment 1:

$$\mu = 0 + (task / target\_c) + (0 + (task / target\_c) \mid sj)$$

and in experiment 2:

$$\mu = 0 + (condition / target\_c) + (0 + (condition / target\_c) \mid sj)$$

Figures **E1** and **E2** show the comparison between the observed data (i.e., the density distribution of the dRTs) and simulated data (100 draws) from the posterior predictive distribution for experiments 1 and 2, respectively. Simulated data (y_{rep}) are the thin, light blue curves and the observed data (y) is the dark blue curve. If the model is a good fit for the data, then the generated data should look similar to the observed data. However, the reverse is not true: the fact that the generated data look similar to the observed data does

not imply that the model is a good fit. Both figures suggest that a normal distribution is a reasonably good approximation for the dRT distributions.

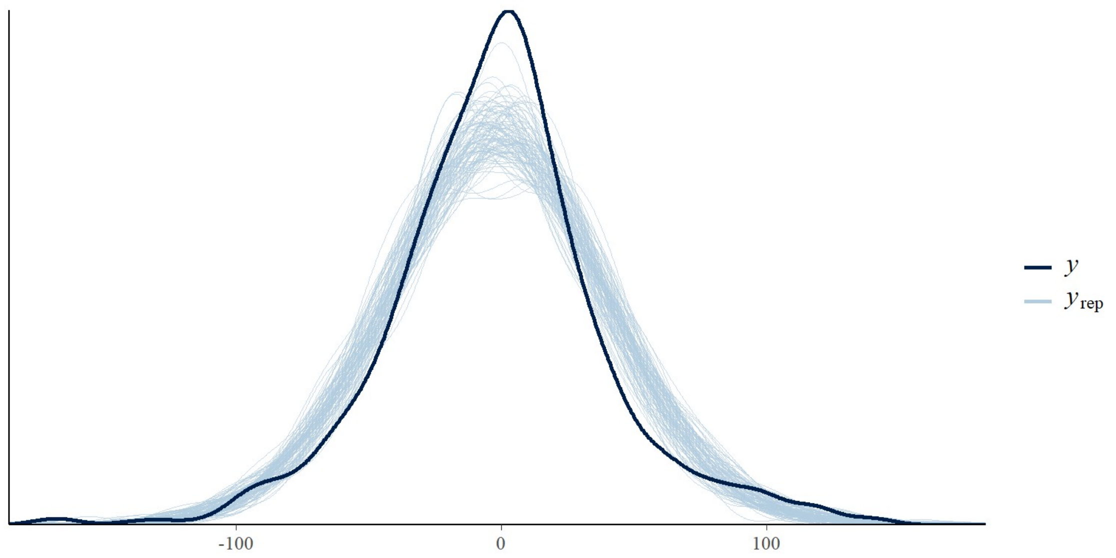

Figure E1: comparison between the observed data (y; i.e., the density distribution of the dRTs) and simulated data (y_{rep}; 100 draws) from the posterior predictive distribution for experiments 1.

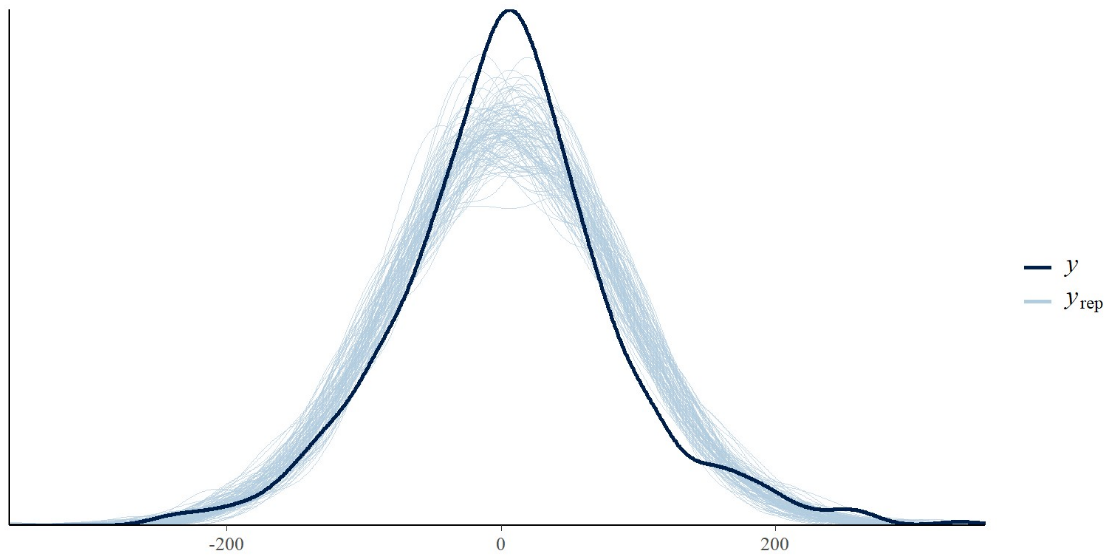

Figure E2: comparison between the observed data (y; i.e., the density distribution of the dRTs) and simulated data (y_{rep}; 100 draws) from the posterior predictive distribution for experiments 2.

Table E1. Response mappings used in the tasks of experiment 1. Response mappings A and B were presented in two different blocks in counterbalanced order across participants. Fourteen participants started all tasks with response mapping A and 11 participants with response mapping B.

	Response mapping	Left key	Right key
Parity task	A	2 4 6 8	1 3 7 9
	B	1 3 7 9	2 4 6 8
Color tasks	A	Dark blue	Light blue
	B	Light blue	Dark blue

Table E2: Secondary task order, cue-target order, and response mappings used in experiment 2. There are 32 combinations, one per participant. P: parity is the secondary task; F: font is the secondary task.

**All blocks**	**Blocks 1**		**Block 2**		**Blocks 3**		**Blocks 4**	
**Left key (color task)**	**Cue-target order**	**Secondary task (go trial)**	**Cue-target order**	**Secondary task (go trial)**	**Cue-target order**	**Secondary task (go trial)**	**Cue-target order**	**Secondary task (go trial)**
Blue	cue-first	P (odd)	target-first	P (odd)	cue-first	F (bold)	target-first	F (bold)
						F (no-bold)		F (no-bold)
		P (even)		P (even)		F (bold)		F (bold)
						F (no-bold)		F (no-bold)
		F (bold)		F (bold)		P (odd)		P (odd)
						P (even)		P (even)
		F (no-bold)		F (no-bold)		P (odd)		P (odd)
						P (even)		P (even)
	target-first	P (odd)	cue-first	P (odd)	target-first	F (bold)	cue-first	F (bold)
						F (no-bold)		F (no-bold)
		P (even)		P (even)		F (bold)		F (bold)
						F (no-bold)		F (no-bold)
		F (bold)		F (bold)		P (odd)		P (odd)
						P (even)		P (even)
		F (no-bold)		F (no-bold)		P (odd)		P (odd)
						P (even)		P (even)
green	cue-first	P (odd)	target-first	P (odd)	cue-first	F (bold)	target-first	F (bold)
						F (no-bold)		F (no-bold)
		P (even)		P (even)		F (bold)		F (bold)
						F (no-bold)		F (no-bold)
		F (bold)		F (bold)		P (odd)		P (odd)
						P (even)		P (even)
		F (no-bold)		F (no-bold)		P (odd)		P (odd)
						P (even)		P (even)
	target-first	P (odd)	cue-first	P (odd)	target-first	F (bold)	cue-first	F (bold)
						F (no-bold)		F (no-bold)
		P (even)		P (even)		F (bold)		F (bold)
						F (no-bold)		F (no-bold)
		F (bold)		F (bold)		P (odd)		P (odd)
						P (even)		P (even)
		F (no-bold)		F (no-bold)		P (odd)		P (odd)
						P (even)		P (even)

Table E3. Response mappings used in experiment 2 in the secondary task. Each response mapping was presented to 8 participants.

Response mapping	Secondary task	Both keys (go)	No keys (no-go)
A	Parity task	odd	even
	Font task	bold	no-bold
B	Parity task	odd	even
	Font task	no-bold	bold
C	Parity task	even	odd
	Font task	bold	no-bold
D	Parity task	even	odd
	Font task	no-bold	bold
